# Transcriptome Profiles Reveal a 12-Signature Metabolic Prediction Model and a Novel Role of Myo-Inositol Oxygenase in the Progression of Prostate Cancer

**DOI:** 10.3389/fonc.2022.899861

**Published:** 2022-05-20

**Authors:** Wangrui Liu, Jianfeng Xiang, Xinrui Wu, Shiyin Wei, Haineng Huang, Yu Xiao, Bo Zhai, Tao Wang

**Affiliations:** ^1^Department of Interventional Oncology, Renji Hospital, Shanghai Jiao Tong University School of Medicine, Shanghai, China; ^2^Department of Clinical Medicine, Medical School of Nantong University, Nantong, China; ^3^ Affiliated Hospital of Youjiang Medical University for Nationalities, Baise, China; ^4^State Key Laboratory of Oncogenes and Related Genes, Shanghai Cancer Institute, Renji Hospital, School of Medicine, Shanghai Jiao Tong University, Shanghai, China

**Keywords:** prostate adenocarcinoma, metabolic prediction models, myo-inositol oxygenase, progression, machine learning

## Abstract

Prostate adenocarcinoma (PRAD) is an extremely common type of cancer in the urinary system. Here, we aimed to establish a metabolic signature to identify novel targets in a predictive model of PRAD patients. A total of 133 metabolic differentially expressed genes (MDEGs) were identified with significant prognostic value. Least absolute shrinkage and selection operator (LASSO) regression analysis was used to construct a 12-mRNA signature model, a metabolic prediction model (MPM), in 491 PRAD patients. The risk score of the MPM significantly predicted the progression of PRAD patients (p < 0.001, area under the curve (AUC) = 0.745). Furthermore, myo-inositol oxygenase (MIOX), the most prominently upregulated metabolic enzyme and hub gene in the protein–protein interaction network of the MPM, showed significant prognostic implications. Next, MIOX expression in normal prostate tissues was lower than in PRAD tissues, and high MIOX expression was significantly associated with disease progression (p = 0.005, HR = 2.274) in 81 PRAD patients undergoing first-line androgen receptor signaling inhibitor treatment from the Renji cohort. Additionally, MIOX was significantly involved in the abnormal immune infiltration of the tumor microenvironment and associated with the DNA damage repair process of PRAD. In conclusion, this study provides the first opportunity to comprehensively elucidate the landscape of prognostic MDEGs, establish novel prognostic modeling of MPM using large-scale PRAD transcriptomic data, and identify MIOX as a potential prognostic target in PRAD patients from multiple cohorts. These findings help manage risk assessment and provide valuable insights into treatment strategies for PRAD.

## Introduction

Prostate adenocarcinoma (PRAD) is primarily a hormone-driven disease mediated by cell growth that is driven by androgen receptor (AR) signaling. Elevated serum concentrations of the AR downstream target prostate-specific antigen (PSA) are evidence in support of this AR-mediated tumor growth. However, the therapeutic effect of castration therapy for PRAD patients is still not known to clinicians (1). The main reason is the presence of an AR amplification, mutation, or splice variant, which can eventually lead to castration-resistant prostate cancer. The prognosis of such patients remains unclear (2). In addition to those “AR-dependent” castration-resistant adenocarcinomas, a subset of patients was found to progress in AR-independent cancer biology, with short-term responses to hormone therapy, early and widespread metastases, and poor outcomes. Notably, this aggressive variant of prostate cancer is frequently associated with low PSA production and therefore cannot be identified by PSA monitoring, which presents a great challenge for clinicians and an extremely poor prognosis for patients (3). Therefore, early screening and diagnosis of PRAD remain challenging.

Numerous studies have identified many factors that may contribute to altered prostate cancer development, but these do not accurately predict tumor aggressiveness (4). Identifying the genomic alterations that cause cancer cells to transition from benign to malignant is critical (5). The genomic alterations observed in this transition include DNA damage repair capacity, telomerase activity, and loss of p53, among others (6, 7).

In recent years, novel immunotherapies represented by programmed cell death-1/programmed cell death-ligand (PD-1/PD-L1) inhibitors have rapidly emerged in the field of PRAD treatment (8), and their efficacy largely depends on interactions with the tumor microenvironment (TME) (9, 10). Accumulating studies have found that the efficacy of immunotherapy and targeted therapy is inseparable from the individual TME (11, 12). Therefore, exploring the underlying mechanisms of the occurrence and development of TME-driven PRAD, improving the efficiency of various existing treatments, and developing models that can accurately predict the disease are critical to advancing the understanding of the biology and developing better treatments (13, 14).

Homeostasis and evolution of the TME are controlled by close connections between all involved cells. This complex interaction often involves extracellular metabolites, which not only constitute a source of energy supply but also act as communication signals between different cellular compartments (15, 16). Cancer cells can use byproducts of sugar metabolism to hijack the functions of tumor-infiltrating immune cells for their benefit. All of these nutrient limitations can shape the metabolism of the developing tumor and thus act as a prominent invasive force (17).

This study aimed to first establish and validate an efficient prognostic metabolic prediction model (MPM) that recruits large-scale transcriptome metabolic genes in PRAD patients. We hypothesized that the MPM classifier could facilitate risk management and treatment strategies for PRAD patients and identify new targets in the combined network of MPMs, providing clinicians with a precise prognostic model for treating PRAD with new insights into precise treatment directions.

## Materials and Methods

### Data Collection

This study used publicly available mRNA expression and clinical data from the PRAD cohort. Consent and ethical approval from registered patients are available in the relevant original article where the dataset was published. A total of 495 PRAD patients from the online dataset were obtained from The Cancer Genome Atlas (TCGA) database (https://portal.gdc.cancer.gov/).

### Identification of Differentially Expressed Genes About Metabolism

Overall, 41 metabolic pathways were selected according to the Kyoto Encyclopedia of Genes and Genomes (KEGG) pathway atlas. The 133 metabolic genes were utilized to identify significant metabolic differentially expressed genes (MDEGs) using the Limma R package (Version 3.6.5) with false discovery rate (FDR) < 0.05 and |logFC| > 0.5.

### Metabolic Prediction Models

Univariate Cox regression analysis was used to identify prognostic implications of significant MDEGs, which were presented in a forest plot using the survival R package (18). Least absolute shrinkage and selection operator (LASSO) regression analysis was performed to construct the 12-mRNA signature model and MPMs in PRAD patients from TCGA cohorts with the glmnet and survival R packages (19).

### Cox Regression Analysis and Receiver Operating Characteristic Curve Construction

All PRAD patients from TCGA cohorts were included for subsequent analysis. Univariate and multivariable Cox regression analyses were used to evaluate the independent prognostic value of the metabolic clusters using a forest plot. The receiver operating characteristic (ROC) curve was constructed for traditional clinical pathologic parameters and the risk score of MPMs in TCGA cohorts. The area under the curve (AUC) was utilized to assess the predictive value of these prognostic signatures.

### Tumor Microenvironment Purity Assessment

The ESTIMATE algorithm was utilized to evaluate total and immune scores using the estimate package (http://r-forge.rproject.org; repos=rforge, dependencies=TRUE) in patients from TCGA cohort. Associations between TME purity and risk score of MPMs or myo-inositol oxygenase (MIOX) expression were assessed using Pearson’s r test.

### Differential Myo-Inositol Oxygenase mRNA Expression and Survival Analysis

Differentially expressed MIOX levels were evaluated between PRAD and normal samples from TCGA cohort using a Student’s t-test. The Kaplan–Meier (KM) method with 95% CIs and a log-rank test was used for survival analysis in the Renji cohort. All analyses were performed in R (Version 4.0.1) and GraphPad Prism 8.0. Results were considered statistically significant when p = 0.05.

## Results

### Identification of Metabolic Differentially Expressed Genes in Both The Cancer Genome Atlas Cohorts

**Figure 1:**
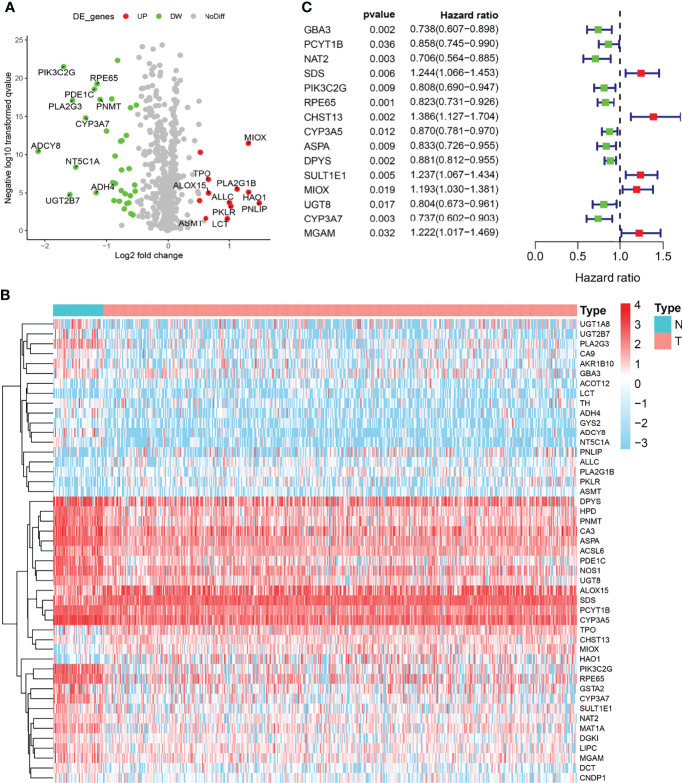
Identification of metabolic differentially expressed genes (MDEGs) in both The Cancer Genome Atlas (TCGA) cohorts. **(A)** Identification of significant MDEGs from 133 metabolic genes. **(B)** Hierarchical partitioning of significant MDEGs was acquired from DNA microarrays based on TCGA cohort. **(C)** Univariate Cox regression analysis of 46 significant MDEGs (p < 0.05) in TCGA cohort was performed in a forest plot.

The expression levels of 133 metabolic genes were collected from 495 PRAD samples in TCGA cohort. Then, these 133 metabolic genes were utilized for further analysis, where 46 significant MDEGs were identified and visualized in a volcano plot (**Figure 1A**). Hierarchical partitioning of significant MDEGs was acquired from DNA microarrays based on TCGA cohort (**Figure 1B**). The mRNA expression levels of these genes were examined across 495 PRAD patients and normal controls, with high expression shown in red and low expression shown in green. Additionally, a univariate Cox regression analysis of 58 significant MDEGs (p < 0.05) in TCGA cohort was performed in a forest plot (**Figure 1C**). Markedly, LASSO regression analysis was used to construct a 12-mRNA signature model and MPMs in PRAD patients of TCGA cohort. By comparing the p-value and hazard ratio, we analyzed the impact of each key gene on the survival of PRAD patients, and we selected MIOX as a hub gene regulating PRAD metabolic disorders (p = 0.019, HR = 1.193).

### Survival Risk Assessment of Metabolic Prediction Models in The Cancer Genome Atlas Cohort

**Figure 2:**
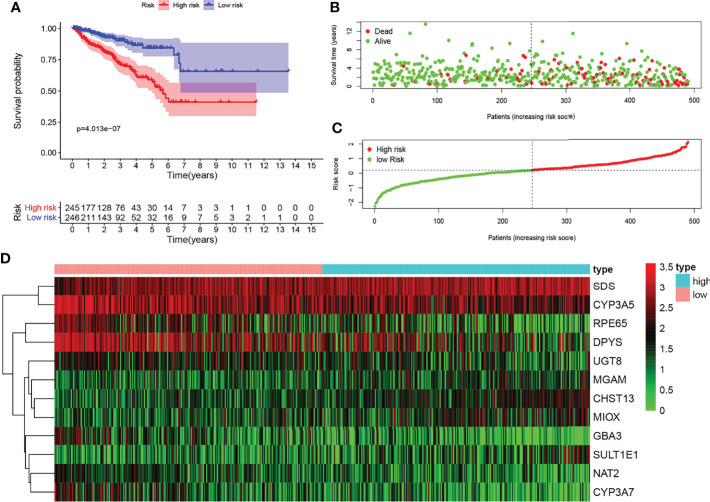
Survival risk assessment of metabolic prediction models (MPMs) consists of a metabolic 12-mRNA signature in The Cancer Genome Atlas (TCGA) and the Clinical Proteomic Tumor Analysis Consortium (CTPAC) cohorts. **(A)** The 12-mRNA signature model (MPMs) in prostate adenocarcinoma (PRAD) patients was calculated using least absolute shrinkage and selection operator (LASSO) regression analysis. Kaplan–Meier survival analysis showed significant predictive value of the risk score depending on MPMs in TCGA cohort. **(B–D)** The distribution of survival time, **(B)** status, **(C)** risk score, and **(D)** hierarchical partitioning of 12 signatures in tumor and normal samples is shown in TCGA cohort.

KM survival analysis showed the significant predictive value of the risk score depending on MPMs in TCGA cohort (**Figure 2A**). The prediction effect of the 12-mRNA signature model is statistically significant for 491 PRAD patients (p < 0.001). The high-risk group is marked in red, and the low-risk group is marked in blue. A survival risk assessment of MPMs consisting of the metabolic 12-mRNA signature was performed. The distributions of survival time, status (**Figure 2B**), risk score (**Figure 2C**), and hierarchical partitioning (**Figure 2D**) of MPMs in tumor and normal samples are shown in TCGA cohort (p < 0.001).

### Cox Regression Analysis, Receiver Operating Characteristic Analysis, and Nomogram of Independent Prognostic Factors and Metabolic Prediction Models in Prostate Adenocarcinoma Patients

**Figure 3:**
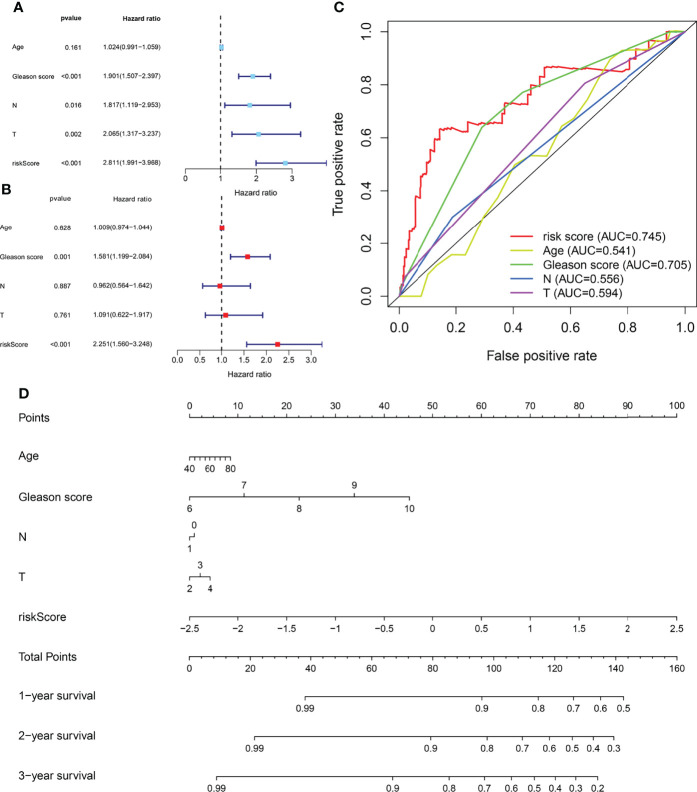
Cox regression analysis, receiver operating characteristic (ROC) analysis, and nomogram of independent prognostic factors and metabolic prediction models (MPMs) in prostate adenocarcinoma (PRAD) patients. **(A, B)** Univariate and multivariate Cox regression analyses enrolling clinical pathologic parameters and MPMs are illustrated in The Cancer Genome Atlas (TCGA) cohort using forest plots. Risk score of MPMs significantly predict prognosis for PRAD patients in TCGA. **(C)** ROC analysis shows robust predictive value of MPMs in TCGA cohort (area under the curve (AUC) = 0.745). **(D)** A nomogram was constructed based on four independent prognostic factors in PRAD patients.

Univariate and multivariate Cox regression analyses enrolling clinical pathologic parameters and MPMs are illustrated using forest plots (**Figures 3A, B****)**. The risk score of MPMs significantly predicts the prognosis for PRAD patients in TCGA (p < 0.001, HR = 2.251). In addition, ROC analysis showed a robust predictive value of MPMs in TCGA (AUC = 0.745) cohorts (**Figure 3C**). A nomogram was constructed based on four independent prognostic factors, including Gleason score, pathologic N stage, pathologic T stage, and risk score of MPMs in PRAD patients (**Figure 3D**).

### Gene Ontology, Kyoto Encyclopedia of Genes and Genomes, and Gene Set Enrichment Analysis

**Figure 4:**
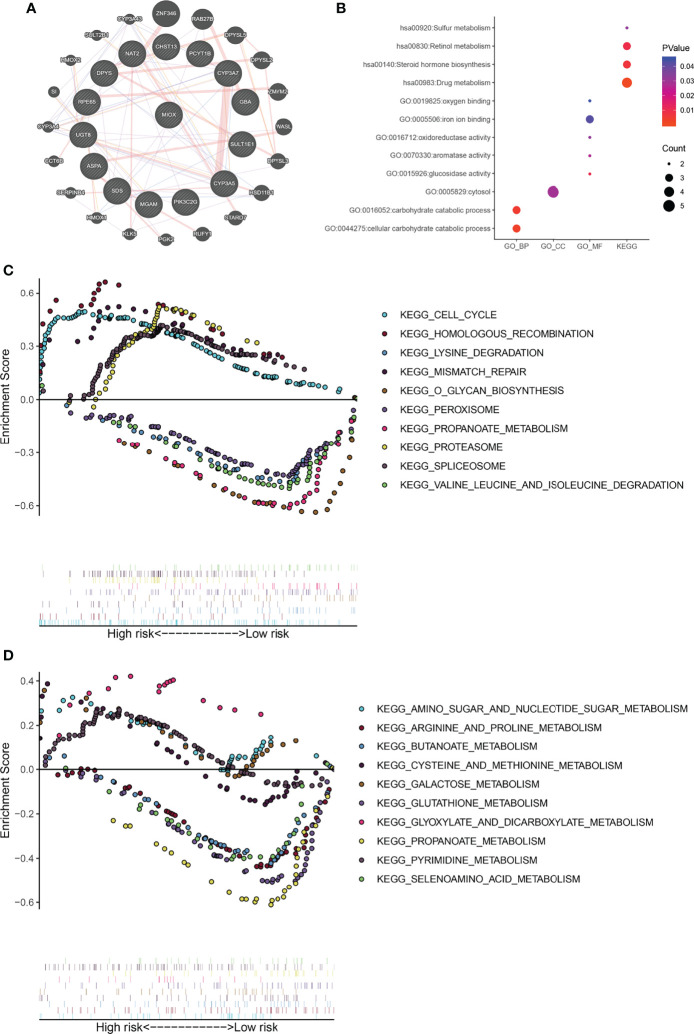
Gene Set Enrichment Analysis (GSEA) indicated significantly altered Gene Ontology (GO) and Kyoto Encyclopedia of Genes and Genomes (KEGG) pathways based on differential risk scores of metabolic prediction models (MPMs) in prostate adenocarcinoma (PRAD) patients. **(A)** A protein–protein interaction (PPI) network was constructed in 12 metabolic mRNA signatures in MPMs. **(B)** GO analysis in high- or low-risk PRAD patients in The Cancer Genome Atlas (TCGA) cohort. **(C)** The top five significantly altered KEGG pathways in high- or low-risk PRAD patients in TCGA cohort. **(D)** The top five significantly altered KEGG pathways in high- or low-risk PRAD patients of the Clinical Proteomic Tumor Analysis Consortium (CTPAC) cohort.

MIOX, a hub gene in the protein–protein interaction (PPI) network of MPMs, shows significant prognostic value in 495 PRAD patients from TCGA cohorts. The PPI network was constructed in 20 metabolic mRNA signatures in MPMs (**Figure 4A**). Gene Ontology (GO) term analysis showed that the genes that were significantly correlated with MIOX are involved in sulfur metabolism, retinol metabolism, and oxygen binding (**Figure 4B**). Gene Set Enrichment Analysis (GSEA) indicated significantly altered KEGG pathways based on differential risk scores of MPMs in PRAD patients with available transcriptomics data from TCGA and Clinical Proteomic Tumor Analysis Consortium (CTPAC) cohorts. The top five significantly altered KEGG pathways in high- or low-risk PRAD patients were examined in TCGA (**Figures 4C, D****)** cohort. We found that the pathways involving the PRAD-related genes are mainly related to the Cell cycle, Homologous recombination, Lysine degradation, Amino sugar and nucleotide sugar metabolism, Arginine and proline metabolism, and Butanoate metabolism. This indicates that MIOX, as a key gene, is involved in the regulation of the cell cycle and metabolic pathways of tumor cells in PRAD.

### Myo-Inositol Oxygenase Promotes an Immune-Infiltrated Tumor Microenvironment and Glycolytic Effects of Prostate Adenocarcinoma

**Figure 5:**
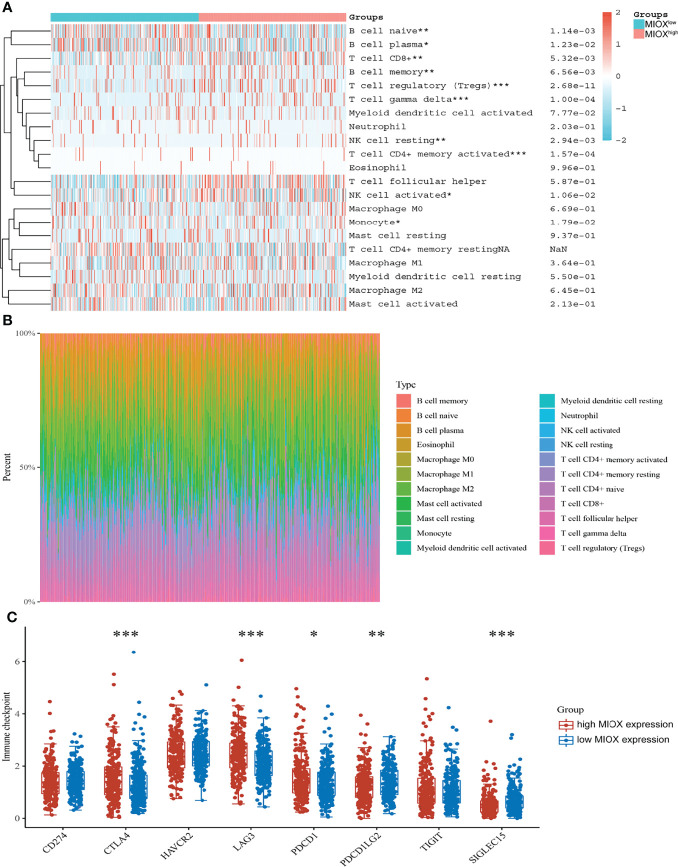
Myo-inositol oxygenase (MIOX) promotes an immune-infiltrated tumor immune microenvironment (TIME) and glycolytic effects of prostate adenocarcinoma (PRAD). **(A)** The CIBERSORT algorithm was performed to characterize the immune cell composition of complex tissues from their gene expression profiles of PRAD in The Cancer Genome Atlas (TCGA). **(B)** The percentage of immune cells in PRAD with high MIOX expression. **(C)** Expression of immune checkpoint molecules was assessed using unpaired t-tests. *p < 0.05, **p < 0.01, ***p < 0.001.

Next, based on the CIBERSORT algorithm, we characterized the immune cell composition of complex tissues using their gene expression profiles of PRAD from TCGA. As shown in **Figure 5A**, we found significant enrichment in T-cell regulatory and T-cell CD4+ memory activated, while decreased naive B cells and myeloid dendritic cells activated in the high MIOX expression group. Next, we examined the percentage of immune cells expressing MIOX-high PRAD, with T cells accounting for a large proportion (**Figure 5B**). Additionally, for patients with higher MIOX expression, the expression levels of most immune checkpoint genes were significantly increased, including CTLA-4, LAG-3, PD-1, PD-L2, and SIGLEC15(**Figure 5C**).

### Elevated Myo-Inositol Oxygenase Expression Predicts Progression in the Renji Cohort

**Figure 6:**
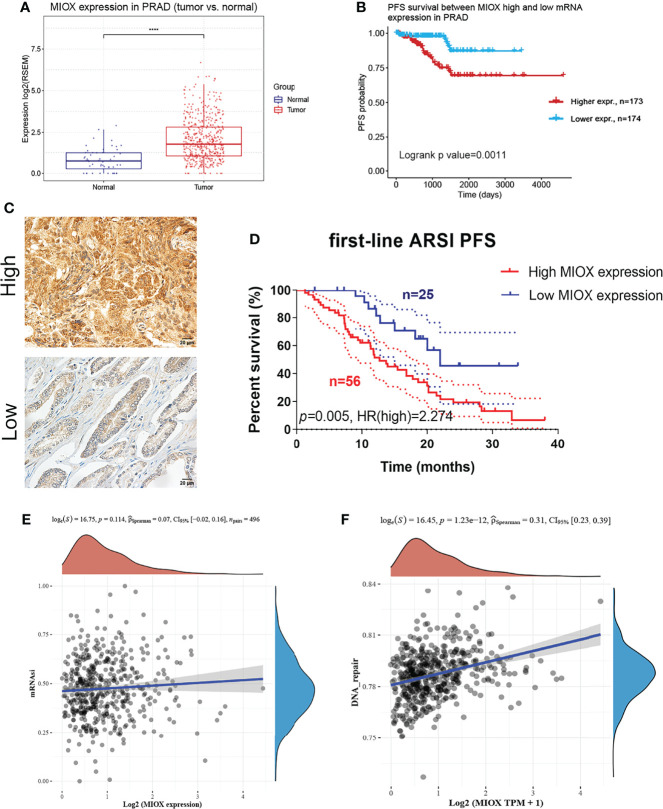
Differential myo-inositol oxygenase (MIOX) expression predicts outcomes in 81 prostate adenocarcinoma (PRAD) patients from Renji cohorts. **(A)** MIOX was expressed at lower levels in normal prostate tissues. **(B)** In PRAD samples from The Cancer Genome Atlas (TCGA) cohort, higher MIOX expression was associated with worse progression-free survival (PFS) in patients. **(C, D)** High MIOX expression was significantly correlated with poor prognosis (p = 0.005, HR = 2.274) in 81 PRAD patients from the Renji cohort. **(E, F)** Dryness Index Score for MIOX in PRAD.

MIOX mRNA expression was examined across PRAD patients and normal controls. The MIOX expression difference between tumor and normal tissues is very significant (**Figure 6A**, p < 0.001). KM survival analysis showed a significant predictive value of the risk score depending on MPMs in TCGA (**Figure 6B**). The high-risk group is marked in red, and the low-risk group is marked in blue. To validate the increased expression of MIOX in PRAD samples compared with normal prostate tissues using immunohistochemistry staining analysis (**Figure 6C**), we first collected samples and explored the prognostic implications of MIOX expression in 81 PRAD patients undergoing first-line AR signaling inhibitor from the Renji cohort. The nuclear MIOX protein expression levels were significantly higher in patients with worse prognoses compared with those with better prognoses in the Renji cohorts (**Figure 6D**). Additionally, the results suggested that increased MIOX protein expression was closely associated with worse PFS (p = 0.005, HR = 2.274). We then calculated mRNAsi, the stemness index score, of MIOX in PRAD. Although its association with mRNAsi was not significant, we found that the association between MIOX and the DNA damage repair process was statistically significant (p < 0.001) (**Figures 6E, F****)**.

## Discussion

Tumors can phenotypically and functionally damage the blood vessels of the original target organ during development, but tumor growth usually requires neovascularization. In turn, the TME typically exhibits some degree of hypoxia, which favors the upregulation of solute carrier family 40 members and apolipoprotein 2, improves iron uptake by malignant cells, and promotes further proliferative activation (20). Moreover, in different *in vivo* mouse tumor models, metabolism leads to DNA damage-induced upregulation of transcript 4, which is also consistent with our study. The abovementioned processes lead to increased oxidative metabolism with a concomitant reduction in glucose uptake, ultimately leading to endothelial hyperactivation, resulting in increased glucose availability in the TME leading to neovascularization and metastasis (21).

The development of precise and accurate predictive biomarkers to clinically benefit prostate cancer patients remains an urgent and unmet clinical need. Promising predictive biomarkers being investigated by our group are precisely associated with reduced sensitivity to endocrine therapy and DNA repair defects (22, 23). However, this has not been without challenges. The significance of MIOX testing can only be fully realized when studies are conducted with active metabolically targeted therapies, converting MIOX from negative to positive predictive biomarkers (24, 25). Second, not all DNA repair deficiencies respond to treatment, with a recent study showing that men with ATM mutations have poorer treatment outcomes as compared with men with BRCA1/2 mutations. Further research is important to determine the best predictive biomarker suite for PRAD to provide the greatest clinical benefit for patients with lethal prostate cancer (26). Elucidating the pathogenesis of DNA repair proteins in prostate cancer could help identify strategies that may have therapeutic benefits, and a metabolic perspective would be even stronger. Taken together, these data demonstrate how detailed studies of protein function can lead to laboratory findings that can potentially impact the management and treatment of prostate cancer.

MIOX is a 32-kDa cytoplasmic enzyme that is expressed in the proximal renal tubule and is upregulated in hyperglycemia (27, 28). A previous study showed that phosphorylation of serine/threonine residues of MIOX can enhance its enzymatic activity (29). Interestingly, the MIOX promoter includes osmotic pressure, carbohydrates, sterols, and oxidative-antioxidative response elements, and thus its transcription is regulated by organic osmotic regulators, high sugar, fatty acids, and oxidative stress (30, 31). We can argue that the upregulation of MIOX is associated with changes in cellular redox, as its promoter contains oxidative response elements. Previous studies have shown that the upregulation of MIOX in acute tubular injury is mediated by oxidants and endoplasmic reticulum stress. The latest research also shows that high blood sugar can lead to increased oxidative and endoplasmic reticulum stress while promoting each other’s activities (32). Therefore, we believe that MIOX plays a key role in the TME of PRAD. As a core gene of aerobic metabolism and glucose metabolism in the TME, it can affect the prognosis of PRAD patients.

There are some limitations of this work. Our study is a single-center study, and in the future, we will conduct a multicenter prospective study to verify the conclusions. In addition, we will conduct *in vitro* and *in vivo* experiments to explore the potentially effective functions of MIOX and reveal the underlying mechanisms.

## Conclusion

Overall, this study comprehensively elucidated the prognostic MDEGs landscape, established novel prognostic MPMs using large-scale PRAD transcriptome data, and identified MIOX as a potential prognostic target in PRAD patients from multiple cohorts. These findings could assist in managing risk assessment and provide valuable insight into treatment strategies for PRAD.

## Data Availability Statement

The original contributions presented in the study are included in the article/**Supplementary Material**. Further inquiries can be directed to the corresponding authors.

## Ethics Statement

Study ethics procedures were approved by Renji Hospital, Shanghai Jiao Tong University School of Medicine.

## Author Contributions

The work was performed in cooperation with all authors. TW, BZ, and YX defined research topics, discussed analysis, supervise studies, provided funding, and revised manuscript. WL, XW, and SW drafted the manuscript, analyzed the data, and interpreted and validated the results. JX, WL, and TW assisted in performing data collection, statistical analysis, and reference collection. XW and WL helped in IHC analysis and patients’ information collection from FUSCC. All authors read and approved the final manuscript.

## Funding

This work is supported by the National Key Research and Development Program: 2020YFC0122305 and General Program from the National Natural Science Foundation of China: 82070619.

## Conflict of Interest

The authors declare that the research was conducted in the absence of any commercial or financial relationships that could be construed as a potential conflict of interest.

## Publisher’s Note

All claims expressed in this article are solely those of the authors and do not necessarily represent those of their affiliated organizations, or those of the publisher, the editors and the reviewers. Any product that may be evaluated in this article, or claim that may be made by its manufacturer, is not guaranteed or endorsed by the publisher.

## Abbreviations

PRAD, prostate adenocarcinoma; CTPAC, Clinical Proteomic Tumor Analysis Consortium; GSEA, Gene Set Enrichment Analysis; ICGC, International Cancer Genome Consortium; KEGG, Kyoto Encyclopedia of Genes and Genomes; MDEGs, metabolic differentially expressed genes; MPMs, metabolic prediction models; OS, overall survival; PFS, progression-free survival; PPI, protein–protein interaction; TCGA, The Cancer Genome Atlas; MIOX, myo-inositol oxygenase; AR, androgen receptor; PSA, prostate-specific antigen; TME, tumor microenvironment; AUC, area under the curve; ROC, receiver operating characteristic.
