# Supplementary material for: Transcriptome Profiles Reveal a 12-Signature Metabolic Prediction Model and a Novel Role of Myo-Inositol Oxygenase in the Progression of Prostate Cancer
Source: Front Oncol. 2022 May 20;12:899861. doi: 10.3389/fonc.2022.899861 (PMC9163567; doi:10.3389/fonc.2022.899861)
Supplement: Supplementary file 1 [file DataSheet_1.docx]

The RNA sequencing data of 495 PRAD patients was obtained from The Cancer Genome Atlas (TCGA, <https://portal.gdc.cancer.gov>) with gene IDs converted from Ensembl ID to gene symbol matrix. The FPKM gene expression profile was measured experimentally using the Illumina HiSeq 2000 RNA Sequencing platform by the University of North Carolina TCGA genome characterization center. Level 3 data was downloaded from TCGA data coordination center, with available clinicopathological and survival data.

In addition, the phenotypic and clinical data of 495 PRAD patients were obtained from TCGA training cohort

A total of 770 genes were downloaded from The nCounter® PanCancer Immune Profiling panel (https://www.nanostring.com/products/ncounter-assays-panels/oncology/pancancer-immune-profiling/) and 758 immune genes were matched in TCGA database for further analysis[1].

1. Cesano, A., *nCounter((R)) PanCancer Immune Profiling Panel (NanoString Technologies, Inc., Seattle, WA).* J Immunother Cancer, 2015. **3**: p. 42.
